# Effect of Nirmatrelvir/Ritonavir (Paxlovid) on Hospitalization among Adults with COVID-19: an EHR-based Target Trial Emulation from N3C

**DOI:** 10.1101/2023.05.03.23289084

**Published:** 2023-05-04

**Authors:** Abhishek Bhatia, Alexander J. Preiss, Xuya Xiao, M. Daniel Brannock, G. Caleb Alexander, Robert F. Chew, Megan Fitzgerald, Elaine Hill, Elizabeth P. Kelly, Hemalkumar B. Mehta, Charisse Madlock-Brown, Kenneth J. Wilkins, Christopher G. Chute, Melissa Haendel, Richard Moffitt, Emily R. Pfaff, N3C Consortium

**Affiliations:** 1 University of North Carolina at Chapel Hill, Chapel Hill, NC, USA; 2 RTI International, Durham, NC, USA; 3 School of Medicine, Public Health, and Nursing, Johns Hopkins University, Baltimore, MD, USA; 4 Patient-Led Research Collaborative, NY, USA; 5 University of Rochester, Department of Public Health Sciences and Department of Economics, Rochester, NY, USA; 6 University of Tennessee Health Science Center, Memphis, TN, USA; 7 National Institute of Diabetes & Digestive & Kidney Diseases, Office of the Director, National Institutes of Health, Bethesda, MD, USA; 8 F. Edward Hébert School of Medicine, Department of Preventive Medicine & Biostatistics, Uniformed Services University of the Health Sciences, Bethesda, MD, USA; 9 University of Colorado Anschutz Medical Campus, Aurora, CO, USA; 10 Stony Brook University, Stony Brook, NY, USA; 11 Consortial acknowledgements appear at the end of the paper

## Abstract

This study leverages electronic health record data in the National COVID Cohort Collaborative’s (N3C) repository to investigate disparities in Paxlovid treatment and to emulate a target trial assessing its effectiveness in reducing COVID-19 hospitalization rates. From an eligible population of 632,822 COVID-19 patients seen at 33 clinical sites across the United States between December 23, 2021 and December 31, 2022, patients were matched across observed treatment groups, yielding an analytical sample of 410,642 patients. We estimate a 65% reduced odds of hospitalization among Paxlovid-treated patients within a 28-day follow-up period, and this effect did not vary by patient vaccination status. Notably, we observe disparities in Paxlovid treatment, with lower rates among Black and Hispanic or Latino patients, and within socially vulnerable communities. Ours is the largest study of Paxlovid’s real-world effectiveness to date, and our primary findings are consistent with previous randomized control trials and real-world studies.

## Introduction

The COVID-19 pandemic has had a profound global impact, with over 761 million cases and 6.8 million deaths as of 21 March, 2023.^1^ This crisis has been met with research and drug development efforts at unprecedented speed, resulting in a number of new treatments aimed at lessening the risk of progression to severe disease. One such treatment, nirmatrelvir/ritonavir (Paxlovid), is a combination protease inhibitor that blocks the replication of SARS-CoV-2. In December 2021, the US Food and Drug Administration (FDA) issued an Emergency Use Authorization (EUA) for Paxlovid, enabling its prescription to high-risk SARS-CoV-2-positive patients aged 12 and older.^2^ The EUA was based on the phase II-III EPIC-HR trial, which reported an 88.9% reduction in the risk of COVID-19-related hospitalization or death among those who received Paxlovid compared to those who received placebo.^3^

Since the EUA issuance, a few studies have assessed the effectiveness of Paxlovid using real-world data. An electronic health record (EHR)-based study in the Kaiser Permanente Southern California health system found that fewer than 1% of patients treated with Paxlovid (*n* = 5, 287) were hospitalized or seen in the emergency department within 5-15 days of the drug being dispensed, though this was not compared with a control group.^4^ Another study leveraging a large repository of Israeli health care data found a 46% reduction in risk of severe COVID-19 outcomes in patients treated with Paxlovid (*n* = 4, 737) when compared with controls, showing a protective effect, but at a lower magnitude than the original EPIC-HR analysis.^5^ A retrospective cohort study in Hong Kong also found that patients treated with Paxlovid (*n* = 4, 921) were at decreased risk of hospitalization, with a weighted hazard ratio of 0.79.^6^ Although these studies offer valuable preliminary evidence of Paxlovid’s real-world effectiveness, there remains a dearth of research specifically aimed at understanding the causal effects of the drug on COVID-19 outcomes with large, representative samples derived from real-world data.

A number of EHR studies have also used real-world data to uncover racial and social disparities in the prescription of various COVID-19 treatments in the United States, including Paxlovid. Prior to Paxlovid’s authorization, multiple studies noted racial and social disparities among SARS-CoV-2-positive patients in access to and treatment with monoclonal antibodies (mAb), with Black and Hispanic or Latino patients less likely than White patients to receive treatment with mAb.^7–9^ A more recent large-scale study of EHR data revealed that those disparities have persisted with Paxlovid; from April through July of 2022, the rate of Paxlovid treatment was 35.8% lower among Black adult patients than White adult patients.^10^

Through the National Institute of Health’s (NIH) National COVID Cohort Collaborative (N3C), we have the opportunity to replicate and expand on these and other recent analyses of Paxlovid efficacy and treatment patterns. We use the target trial emulation (TTE) framework and a large, geographically and demographically diverse cohort from N3C’s EHR data repository.^11^ Here, we characterize the population prescribed Paxlovid, assess potential disparities in Paxlovid prescription, and estimate the causal effect of Paxlovid use on hospitalization among SARS-CoV-2-positive adults in the United States.^11^

## Results

**Fig. 1:**
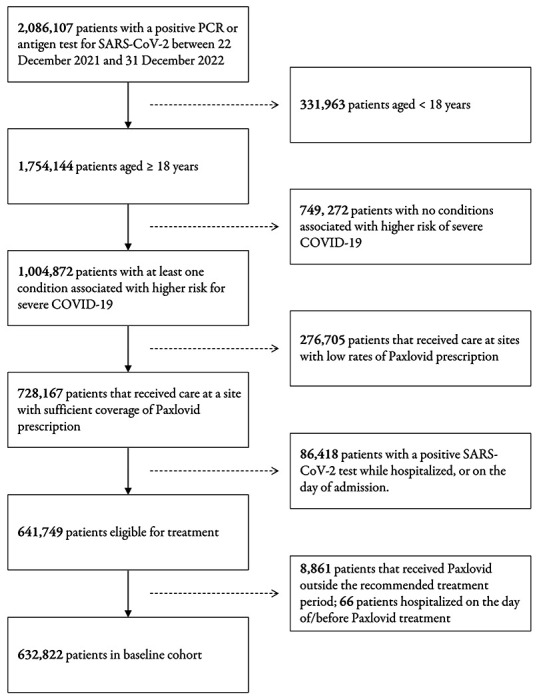
Study Cohort and Flow of Emulated Trial

The hypothesized target trial protocol is articulated in Table 1, and we emulate each component to define our base population within the N3C database (See Figure 1).

### Patient Characteristics

In the unmatched cohort (within our defined study period), a total of 632,822 patients had a valid COVID-19 index date during the study period, of which 111,443 (17.6%) were treated with Paxlovid, and 698 (0.6%) were hospitalized. After applying the eligibility criteria to the patient population and study sites, a total of 33 of 76 study sites were retained. Patients were matched across treatment groups by characteristics selected *a priori* associated with the treatment assignment and outcome, yielding an effective sample size of 410,642 across 33 sites, balanced across all covariates. The characteristics of all patients during the study period are presented in Table 2, stratified by treatment group.

**Fig. 2:**
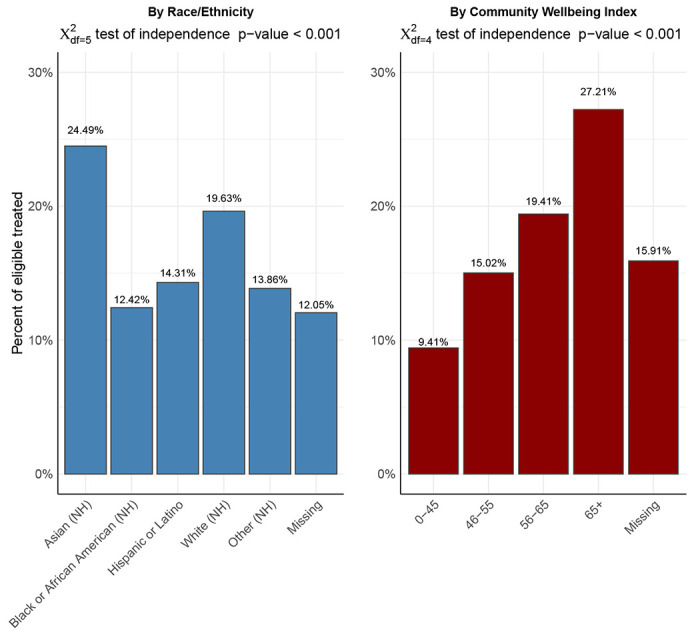
Proportion of individuals in base population stratified by Race/Ethnicity, and ZIP Code-level Community Wellbeing Index (CWBI) Notes: Abbreviations: NH = Non–Hispanic; Lower CWBI scores indicate higher community–level social vulnerability

Among the unmatched cohort, there were large, statistically significant differences in assignment to Paxlovid treatment - 12.42% of Black, non-Hispanic patients and 14.31% of Hispanic patients were treated with Paxlovid, compared to 19.63% of White, non-Hispanic patients, and 24.49% of Asian, non-Hispanic patients (*χ*^2^_*df*=5_ test of independence p-value < 0.001). When stratified by patients’ residential areas, patients who lived in areas with higher Community Wellbeing Index (CWBI) values (and lower corresponding social vulnerability) were also more likely to be treated with Paxlovid. (*χ*^2^_*df*=4_ test of independence p-value < 0.001). (See Figure 2)

### Effect of Paxlovid on Hospitalization

In the unmatched cohort, 698 (0.6%) patients treated with Paxlovid were hospitalized in the follow-up period, compared to 10,673 (2.0%) patients in the untreated group. In the primary analysis using the matched cohort, patients treated with Paxlovid had lower unadjusted causal odds of hospitalization compared with patients not treated with Paxlovid (OR, 0.33; 95% CI, 0.24-0.45), and this was consistent with the estimate adjusted for potential confounders (OR, 0.35; 95% CI, 0.29-0.42). (See Table 3)

The adjusted estimates in the primary analysis adjust for the effects of CWBI captured at the patient level, including patients that were categorized as “Missing” for any CWBI data, and we assume that missingness was unrelated to unmeasured confounding or treatment assignment. Results of our sensitivity analysis showed that the estimates for our primary analysis were robust against missing CWBI data, both when sites were dropped (OR, 0.36; 95% CI, 0.29-0.44), or when the covariate itself was dropped (OR, 0.37; 95% CI, 0.31-0.45). Additionally, sensitivity analysis aimed at identifying the potential effects of immortal time bias also showed that our estimates were stable (OR, 0.38; 95% CI, 0.31-0.46). (Supplementary Table 1).

### Effect adjusted by COVID-19 Vaccination Status

For our subgroup analysis estimating the effect of Paxlovid on hospitalization adjusting for the effect of vaccination, individuals from nine sites with trusted vaccine data were included. A total of 170,063 individuals included in the base population were vaccinated, of which 45,929 (27.0%) received Paxlovid, and 1,873 (1.1%) were hospitalized; 51,306 individuals were unvaccinated, of which 6,633 (12.9%) received Paxlovid , and 1,152 (2.2%) were hospitalized. After CEM matching (*n* = 136, 815), weighting, and adjusting for both the main effects and interaction effects of vaccination status, this analysis was consistent with the primary results, and patients prescribed Paxlovid had lower adjusted causal odds of hospitalization (OR, 0.32; 95% CI, 0.24-0.42). Vaccinated patients also had lower adjusted causal odds of hospitalization (OR, 0.49; 95% CI, 0.41-0.58). Additionally, there was no significant evidence of an interaction effect between the effect of Paxlovid and vaccination on hospitalization at 28 days, suggesting no heterogeneity by vaccination status in the effect of Paxlovid treatment (OR, 1.08; 95% CI, 0.73-1.61).

## Discussion

Despite the widespread use of nirmatrelvir/ritonavir (Paxlovid) for the treatment of COVID-19, we are still in the early stages of understanding its protective effect at scale in real-world settings. In this target trial emulation using the N3C database, one of the largest longitudinal observational datasets on COVID-19 patients in the United States, individuals treated with Paxlovid within five days of a COVID-19 diagnosis or positive SARS-CoV-2 test had 65% lower odds of hospitalization, compared with those never treated with Paxlovid. When accounting for vaccination status, individuals treated with Paxlovid had a 68% lower odds of hospitalization, and this effect did not depend on an individual’s vaccination status. We also find significant differences in distribution of Paxlovid across race, ethnicity, and social vulnerability strata. Paxlovid is thus effective at lessening the risk of hospitalization, but is not equitably distributed.

We examined the distribution of Paxlovid treatment by race and ethnicity, along with a measure corresponding to a patient’s residential ZIP Code-level community well being index (CWBI). We found large differences in Paxlovid treatment rates by race/ethnicity and CWBI. Across race/ethnicity strata, Black and Hispanic/Latino patients were on average less likely to receive Paxlovid treatment, compared to White and Asian patients. Additionally, patients from communities with higher levels of social vulnerability (lower CWBI values) were also less likely to receive Paxlovid treatment. Our findings are consistent with prior research from December 2021- July 2022 that also documented disparities in outpatient oral antiviral treatment by both ZIP Code-level social vulnerability and race/ethnicity, and showed that Black patients had 35.8% lower rates of treatment compared to White patients, and Hispanic patients had 29.9% lower rates of treatment compared to non-Hispanic patients. These disparities in access to treatment are of particular concern due to the growing evidence base that shows that individuals in these groups are more likely to experience higher levels of COVID-19 exposure, discrimination in access to care, and severe COVID-19 health outcomes. ^12–17^ The reasons for these disparities are a complex constellation of factors including (and not limited to) a history of systemic discrimination and racism in treatment, lack of physical and economic resources to facilitate equal access in vulnerable communities, lack of information and knowledge about treatment options, and language barriers owing to a lack of culturally competent care.^10^ Without attention, recognition, and remediation on the part of providers, public health agencies, the health system, and communities, the disproportional burden of COVID-19 will only further exacerbate existing health inequities in the United States.

Findings from our primary analysis were consistent with existing randomized control trial results. The EPIC-HR randomized control trial (*n* = 2, 246) among symptomatic, non-hospitalized, high-risk patients assessed the effect of Paxlovid on hospitalization or death through a 28-day follow-up period, and found a 89.9% risk reduction.^4^ The lower magnitude of effect in our findings may be attributable to differences in study design (as noted by Najjar-Debbiny et. al): 1) differences in dominant strains at the time of the study; 2) their inclusion of only symptomatic patients versus our inclusion of all COVID-19 positive patients (inclusive of asymptomatic patients); 3) their earlier treatment assignment within five days from symptom onset versus our treatment assignment within five days of a COVID-19 diagnosis or a laboratory-confirmed SARS-CoV-2 test; and 4) our intention-to-treat approach with no information on adherence to treatment, compared to their clinical trial setting.^5^ We also adjusted for the effects of vaccination, and interestingly, we found that the effects of treatment with Paxlovid were not heterogeneous across vaccinated and unvaccinated individuals. This finding is of particular interest given the preliminary evidence from the EPIC-SR trial that the impact of Paxlovid on vaccinated populations was uncertain.^18^ Further research is needed on the effectiveness and cost-effectiveness of Paxlovid treatment in these populations. It is also worth noting that the omission of vaccination status did not significantly bias our estimates in our primary analysis.

Our findings are also consistent with other real-world analyses suggesting a protective effect of Paxlovid. Najjar-Debbiny et. al applied Cox hazard models and found that treatment with Paxlovid resulted in a 46% reduced risk of severe COVID-19 and 80% reduced risk of mortality in Israel, with no evidence for the interaction effect of Paxlovid and vaccination (*n* = 180, 351).^5^ While their outcome of interest was severe COVID-19 or death, ours examined hospitalization, complementing their findings and notably, expanding on their regression-adjusted approach by including a causal inference framework through target trial emulation to estimate the causal effect of Paxlovid. Our estimates also support results from a retrospective cohort study in Hong Kong (*n* = 93, 833), which estimated a propensity score-weighted 21% reduction in hospitalization risk among patients treated with Paxlovid.^6^ However, while this analysis used population-level vaccination coverage as an estimate for patient-level vaccination status, our sub-analysis matched patients on their health-record documented vaccination information to provide a more precise estimate among vaccinated populations.

### Strengths

This study has several strengths that underscore the value of large-scale EHR repositories for advancing comparative effectiveness research. In the absence of large-scale RCTs, target trial emulations allow researchers to explore treatment effects in real-world settings and identify the most effective treatments for a variety of health conditions. This study is one of a few studies that apply methods to emulate hypothetical target trials, accounting for the effect of confounding.^19–22^ The estimated effects were also consistent across sensitivity analyses targeted at addressing issues in both missingness and potential immortal time bias. Additionally, the analyses were conducted using a large, comprehensive database of EHR data from 33 contributing sites across the United States, increasing generalizability, and decreasing the potential for issues that typically arise from misclassification in administrative or claims data.^23^ The volume of data contained in the N3C database allowed for coarsened exact matching while preserving statistical power, instead of weighting approaches that may be sensitive to model misspecification and the potential for extreme weights leading to biased estimates.^24;25^

### Limitations

This study also has a few limitations. First, since the data did not include any further information on the dose received over the study period (Paxlovid is typically given orally twice daily for five days), we assume complete adherence to treatment and no loss-to-follow-up, such that the intention-to-treat effect is equal to the per-protocol effect. Second, the sub-analysis on vaccinations did not include individuals with incomplete courses of vaccination (1 dose), nor information on the timing of vaccination relative to COVID-19 infection, and therefore we were unable to shed light on whether the response to Paxlovid varied by additional strata of vaccination. Third, the outcome of interest was a discrete measure of hospitalized/not hospitalized, and further studies may benefit from taking a time-to-hospitalization approach and modeling the effectiveness of Paxlovid using a Cox proportional hazards model, which may further inform smaller differences in treatment-response. Due to the many possible patterns over time of COVID-19 index, Paxlovid treatment, and hospitalization, determining time zero (*t*_0_) in this study was not trivial. We opted to treat hospitalization as a discrete outcome in our follow-up period to simplify the study’s concept of time. Related to the difficulty in establishing *t*_0_, while our sensitivity analysis aimed to demonstrate that our estimates would be consistent even in the presence of substantial immortal time bias, further sensitivity analyses using a nested target trial emulation framework may be useful in substantiating this.^26;27^ Fourth, it is well-documented that EHRs are susceptible to missing data when patients do not seek care, care is provided outside of the reporting facility, or a condition is documented outside of the structured EHR (e.g., in clinical notes), and it is likely that our estimates may be biased if missingness was related to any residual unobserved confounding.^28–30^ We took several steps to mitigate the risk of missing data – all individuals in our cohort have established care at the partner facility both before and after their acute COVID-19 event, as evidenced by documented healthcare encounters, and our vaccination sub-analysis is limited to facilities with a high recorded vaccine ratio to reduce the number of individuals misclassified as unvaccinated. Fifth, our inclusion criteria of Paxlovid treatment within five days of COVID-19 index differs from the indication of treatment within five days of symptom onset. However, we note that within our base cohort, 95.70% of treated patients were treated within one day of COVID-19 index. Finally, our study is subject to the assumptions of all causal inference studies: consistency, positivity, and exchangeability. In particular, the assumption of exchangeability rests on the assumption that there are no unmeasured confounders.

## Conclusion

Among patients with COVID-19 in our study period, the odds of hospitalization within a 28-day follow-up period was 65% lower in patients treated with Paxlovid within five days of COVID-19 index, compared with patients who were never treated with Paxlovid. Although there remains the potential for unmeasured confounding, the results of our large-scale study using EHR data are consistent with the evidence base of smaller-scale RCTs and smaller real-world data studies across other geographies. Our results demonstrate the potential for further research using the target trial emulation framework with observational data to supplement clinical trials. We also found disparities in the rates of Paxlovid treatment. Black patients, Hispanic or Latino patients, and patients living in more vulnerable communities were treated with Paxlovid at a significantly lower rate than others. Taking action to remediate these disparities will equalize the opportunity for all high-risk patients to prevent severe COVID-19 outcomes.

## Methods

We performed a target trial emulation to assess the effect of Paxlovid treatment within five days of COVID-19 index on the risk of hospitalization within 28 days of COVID-19 index (see Eligibility Criteria). We followed a two-step process for emulating target trials with observational data: first, we articulated the causal question of interest in the form of a hypothetical randomized trial protocol, specifying eligibility criteria, treatment strategies, treatment assignment, the study period for follow-up, the outcome of interest, causal contrasts, and the analysis plan to estimate effects.^33^ Second, we emulated each component of this protocol using patient-level data inside the NIH-hosted N3C Secure Data Enclave, which integrates EHR data for 18 million patients across 76 participating sites across the United States. N3C’s methods for patient data acquisition, ingestion, and harmonization have been reported in detail elsewhere.^34–36^ All analyses and results as part of this study are reported in adherence with the Strengthening the Reporting of Observational Studies in Epidemiology (STROBE) reporting guidelines.^37^

### Eligibility Criteria

We defined our study period as December 23, 2021, the day after FDA authorization of Paxlovid, to December 31, 2022. To meet the eligibility criteria for the study as per the target trial protocol, we specified the following inclusion criteria: 1) having a documented COVID-19 index date within the study period (with index date defined as the earliest date of either (a) a COVID-19 diagnosis or (b) a positive SARS-CoV-2 test result), 2) being ≥ 18 years of age as of the COVID-19 index date, 3) having one or more risk factors for severe COVID-19 as per CDC guidelines, including age ≥ 50 years old, or the presence of underlying medical conditions associated with a conclusive higher risk of severe COVID-19.^31^ We excluded all patients < 18 due to the potential for differences in both clinical characteristics and prescription practices among pediatric and adult patients prescribed Paxlovid.^38;39^

Additionally, since we were interested in quantifying the causal effect of Paxlovid treatment on the outcome of hospitalization, we applied three exclusion criteria to exclude: 1) patients who were hospitalized on or before the COVID-19 index date or date of treatment with Paxlovid (outcome precluding treatment), 2) patients who received Paxlovid before their COVID-19 index date, and 3) patients who received Paxlovid after the recommended five days following their COVID-19 index date. In order to ensure that data were captured from sites with high fidelity and adequate coverage, we only included data from sites with at least 1% of eligible patients, and a minimum of 100 patients, treated with Paxlovid during the study period.

### Defining Treatment and Outcome

Eligible patients were categorized by their treatment exposure, defined as having been treated with Paxlovid within five days of their COVID-19 index date, with controls defined as patients never treated with Paxlovid. We selected an eligibility window of five days in adherence with recommended clinical guidelines, and to minimize heterogeneity and potential for indication bias. For patients who were never treated with Paxlovid, we used the date of their earliest indication of COVID-19 (diagnosis or positive lab result) within the study period as the index date. For those in the treatment group, we used their earliest indication of COVID-19 within five days of their first Paxlovid treatment date in the study period. As per our exclusion criteria, we excluded patients that received Paxlovid after the 5-day eligibility period. Within the N3C enclave, the “Paxlovid or nirmatrelvir” concept set was used to identify drug exposures that correspond to Paxlovid (10 Observational Medical Outcomes Partnership [OMOP] concepts).^40^ We followed patients for a 28-day period following their COVID-19 index date. Our primary outcome of interest was hospitalization at any point in time during the 28-day follow-up period, specified as a discrete measure corresponding to any hospitalization (*Y* = 1) or no hospitalization (*Y* = 0). For the purposes of this analysis, we did not consider time-to-hospitalization, severity at the time of admission, or any downstream outcomes such as in-hospital death or discharge.

### Statistical Analysis

#### Overview

First, we applied a two-sided Chi-squared test to examine the distribution of Paxlovid treatment across two covariates: 1) patient race/ethnicity, and 2) a ZIP Code-level Community Wellbeing Index (hereafter referred to as CWBI). The CWBI measure is a composite index of social determinants of health available within the N3C database, with higher CWBI values corresponding to a higher level of protective community-level social determinants of health. The index methodology was developed by Sharecare and the Boston University School of Public Health. CWBI values are derived from the patient’s residential ZIP Code-level data across five key inter-related community-level domains: healthcare access (ratios of healthcare providers to population), resource access (libraries and religious institutions, employment, and grocery stores), food access (access to grocery stores and produce), housing and transportation (home values, ratio of home value to income, and public transit use), and economic security (rates of employment, labor force participation, health insurance coverage rate, and household income above the poverty level).^32^

Next, we used a potential outcomes framework to compare the rate of hospitalization among patients who received Paxlovid during the five days following a positive SARS-CoV-2 test to those who did not. To adjust for confounding and emulate random assignment in the context of the target trial, we matched individuals in the treatment and control cohorts on a set of pre-treatment variables: sex, age (binned), race and ethnicity, Charlson Comorbidity Index (CCI) value (as a proxy for all underlying medical conditions; binned), CWBI (binned), month of COVID-19 onset, and site of care provision, including all data present in their electronic health record as of their positive SARS-CoV-2 test. Due to the prevalence of literature suggesting disparity in treatment assignment and outcomes by race, ethnicity, and other social determinants of health, we include these measures as potential confounders.^7–10^ Sex, age, and comorbidities are known to affect both care seeking for and the outcome of COVID-19. The index month was included because Paxlovid treatment rates, viral variants, and infection rates changed during the study period. CCI was coded as missing when no condition exposures were present in N3C prior to index. CWBI was coded as missing when patient ZIP Code was not reported.

We matched patients in the treatment and control groups using coarsened exact matching (CEM) and weighted observations by their CEM weights. CEM weights were defined as:

$$W=\{\begin{array}{cc} 1 & \text{for treated units}\\ \left(\frac{m_{C}}{m_{T}}\right)W_{s} & \text{for control units} \end{array}$$

where *m_C_* and *m_T_* are the numbers of control units and treated units in the sample, $m_{T}^{s}$ and $m_{C}^{s}$ are the number of control units and treated units in stratum *s*, and $W_{s}=\frac{m_{T}^{s}}{m_{C}^{s}}$ .

CEM offers several advantages over other matching methods.^41^ Furthermore, when the likelihood of treatment is heavily determined by the covariates (as in this study), weighting methods like inverse probability of treatment weighting tend to assign extreme weights to treated patients for whom treatment was very unlikely (and vice versa). CEM avoids this issue. Bins used to coarsen covariates were based on subject matter knowledge of the measurement scale of each variable (e.g., age groups based on strata with known differences in COVID-19 outcomes). We examined the results of the specified coarsened exact matching to ensure that it yielded a balanced cohort with a sufficiently large effective sample size.

#### Estimation

Our primary analysis aims to estimate the total effect of receiving treatment with Paxlovid within five days of COVID-19 index date (treatment), compared to not receiving Paxlovid (control), on the discrete outcome of hospitalization.

The unadjusted average treatment effect on the treated (ATT) was first estimated using a difference-in-means estimator:

$$\hat{E\left[\tau_{i}\right]}=\frac{1}{n_{1}}\underset{i:T_{i}=1}{\sum }Y_{i}-\frac{1}{n_{0}}\underset{i:T_{i}=1}{\sum }Y_{i}$$

The unadjusted causal odds ratio among the matched population was then estimated as:

$$\tau_{OR}=\frac{\tau_{\text{treated}}}{\tau_{\text{control}}}$$

The adjusted treatment effect was calculated using a CEM-weighted mixed-effects logistic regression model with a discrete response variable corresponding to hospitalization (*Y* = 1, *Y* = 0), controlled for independent pre-treatment variables. We include site-specific random intercepts to account for within-cluster homogeneity in outcomes and to estimate the influence of the cluster on the outcomes of the individuals within the cluster. We assume that the fixed effects are invariant across clusters and therefore do not include random slopes in the model.

The final CEM-weighted mixed-effects logistic regression model is specified as:

$$g(P\left(Y_{ij}=1\right)=\gamma_{0}+X_{ij}\gamma_{X}+Z_{j}\gamma_{Z}+U_{0j}$$

where $\gamma_{0}$ is the intercept, $\gamma_{X}$ are the effects of the covariates at the individual level, $\gamma_{Z}$ are the effects of the observed covariates at the cluster level, and *g* denotes the logit link function. The cluster-level random intercepts *U*_0*j*_ are assumed to have and are uncorrelated with the included covariates; these induce a data-partner-specific interpretation desired for odds ratios.^42^

#### Vaccination-adjusted Subanalysis

Using the same method applied to the primary analysis described above, we also conducted a subanalysis with COVID-19 vaccination status included as a covariate. We considered this important for two reasons. First, we hypothesized that vaccination status may be a confounder of Paxlovid treatment and hospitalization, largely through the latent variable of infection severity. If so, our primary analysis would violate the assumption of no unmeasured confounding. Second, we hypothesized that Paxlovid treatment may be less effective among vaccinated patients. There is mixed evidence for this dynamic. Pfizer’s EPIC-SR trial found no significant treatment effect among vaccinated patients with one risk factor for severe COVID-19.^43^ A large health system found that Paxlovid treatment further reduced the likelihood of hospitalization and death among vaccinated patients, but that the treatment effect was smaller among fully vaccinated patients.^18^

For this subanalysis, we used a modified cohort of patients from sites with reliable information on patient vaccination status. Vaccination status in N3C is subject to misclassification, particularly among patients who were vaccinated outside of data partner systems. We determined the subset of sites with reliable patient-level vaccination records by the ratio of two statistics: (1) the proportion of individuals who are documented as vaccinated in their EHR and (2) the proportion of individuals who are truly vaccinated. We calculated the first statistic from the EHR data for each site. We estimated the second statistic using CDC-reported vaccination rates for the counties served by each partner facility.^44^ CDC vaccination rates by county are included as a data asset in N3C. Patient counties were inferred using a county-ZIP crosswalk. Each patient’s likelihood of vaccination was drawn from their county’s vaccination rate and the overall expected vaccination rate for a partner facility was computed as the mean of their patients’ vaccination likelihood. We defined a facility’s recorded vaccine ratio as the ratio of these two statistics and limited the vaccination sub-analysis to individuals from facilities with a ratio of at least 0.66.^45^

Accordingly, we categorized patients by their vaccination status prior to their COVID-19 index date, defined as having completed a full course of vaccination at least 14 days prior to index. Partially vaccinated patients and patients who were fully vaccinated fewer than 14 days prior to index were excluded from the analysis. With treatment, follow-up period, and outcomes consistent with the specification of the primary analysis, we re-estimated the treatment effect of Paxlovid on hospitalization using the previously specified mixed-effects logistic regression model, this time with the additional adjustment for the independent effect of vaccination status, and the interaction effect of vaccination and treatment with Paxlovid.

#### Sensitivity Analysis

We conducted two sensitivity analyses with regard to our primary analysis. First, we assessed the sensitivity of our estimated treatment effects to the CWBI as a matching variable, as well as the potential impact of missing CWBI data on treatment effect estimates (if the missingness was related to unmeasured confounders or treatment assignment). CWBI was the only covariate with an overall missing rate (27.40%) higher than 10 percent. CWBI missingness was largely determined by site. Six sites do not report patient ZIP Code data to N3C. An additional four sites had missing ZIP Code data for more than 10 percent of patients. To test for sensitivity to this, we removed data from all sites with a 10% or higher missingness rate for the CWBI covariate, then repeated the matching process and re-estimated the treatment effects on the outcome. In addition, we also repeated the analysis by excluding the CWBI variable entirely from both the matching and estimation processes and reported the resulting adjusted effect estimates. Second, we aimed to understand the potential immortal time bias that may be present in our primary analysis. In our primary analysis, we define time zero (*t*_0_) as the date of a patient’s COVID-19 index date. We excluded all patients who received Paxlovid before t0, and those who were hospitalized between t0 and time of treatment assignment (*t_A_*) (prescription of Paxlovid). This period, a patient’s “immortal time”, may bias estimates in favor of a lower risk in the treatment group, even in the absence of a true effect.^26^ To quantify the bias due to the presence of immortal time, in this sensitivity analysis we included treated patients who were hospitalized on or between *t*_0_ and *t_A_*. This represents a “worst-case scenario”, and provides a conservative estimate of the treatment effect taking into account the potential immortal time bias.

## Supplementary Material

1

## Figures and Tables

**Table 1:** Protocol of a Target Trial to Estimate the Effect of Paxlovid on the rate of hospitalization in the 28-days following a positive SARS-CoV-2 test

Protocol Component	Description under Target Trial Conditions	Method of Target Trial Emulation in this study
Eligibility criteria	Persons aged 18 and older who have one or more risk factors for severe COVID-19 as per CDC guidelines and who have had an COVID-19 infection during the study period.^31^	Persons aged 18 and older who have one or more risk factors for severe COVID-19 as per CDC guidelines and who have had either a documented COVID-19 diagnosis, or laboratory confirmed positive SARS-CoV-2 test during the study period.^31^
Treatment strategies	Complete course of treatment with Paxlovid within five days of symptom onset.	Complete course of treatment with Paxlovid within five days of the date of laboratory-confirmed SARS-CoV-2 test, or COVID-19 diagnosis.
Assignment procedures	Participants will be randomly assigned to treatment or control at baseline and will be aware of their treatment assignment.	Coarsened exact matching on pre-treatment covariates to ensure exchangeability of treatment and control groups and emulate random assignment.
Follow-up period	28 days following a positive SARS-CoV-2 test	28 days following either positive SARS-CoV-2 test, or COVID-19 diagnosis
Outcome	Hospitalization within follow-up period	Hospitalization within follow-up period
Causal contrasts	Intention-to-treat effect	Intention-to-treat effect
Analysis plan	Intention-to-treat effect estimated via comparison of hospitalization rates among individuals assigned to each treatment arm, adjusting for pre-treatment covariates. Additional analyses to include the main and interaction effects of vaccination.	Coarsened exact matching on pretreatment covariates; Intention-to-treat effect estimated via comparison of hospitalization rates among individuals in each treatment arm in the matched cohort, adjusting for pre-treatment covariates. Additional analyses to include the main and interaction effects of vaccination.

**Table 2:** Baseline Population Characteristics Before and After Matching

	Before Matching		After Matching	
Characteristic	No Paxlovid (N= 521,379)	Paxlovid (N=111,443)	No Paxlovid (N=306,132)	Paxlovid (N=104,510)
Hospitalization^1^				
No	510,706 (98.0%)	110,745 (99.4%)	300,583 (98.2%)	103,882 (99.4%)
Yes	10,673 (2.0%)	698 (0.6%)	5,549 (1.8%)	628 (0.6%)
Sex				
Female	315,078 (60.4%)	67,597 (60.7%)	189,642 (61.9%)	63,914 (61.2%)
Male	206,198 (39.5%)	43,829 (39.3%)	116,490 (38.1%)	40,596 (38.8%)
Missing	103 (0.0%)	<20 (0.0%)	-	-
Age (in years)				
18-24	15,963 (3.1%)	1,429 (1.3%)	3,757 (1.2%)	1,018 (1.0%)
25-34	35,194 (6.8%)	4,538 (4.1%)	11,921 (3.9%)	3,657 (3.5%)
35-49	70,172 (13.5%)	12,716 (11.4%)	34,950 (11.4%)	11,172 (10.7%)
50-64	215,304 (41.3%)	42,112 (37.8%)	127,144 (41.5%)	40,101 (38.4%)
65+	184,746 (35.4%)	50,648 (45.5%)	128,360 (41.9%)	49,562 (47.4%)
Missing	-	-	-	-
Race/Ethnicity^2^				
Asian (NH)	11,082 (2.1%)	3,594 (3.2%)	3,393 (1.1%)	2,544 (2.4%)
Black or African American (NH)	74,907 (14.4%)	10,627 (9.5%)	27,638 (9.0%)	9,457 (9.0%)
Hispanic or Latino (Any Race)	49,908 (9.6%)	8,332 (7.5%)	18,638 (6.1%)	7,021 (6.7%)
White (NH)	335,385 (64.3%)	81,891 (73.5%)	235,601 (77.0%)	80,081 (76.6%)
Other (NH)	5,716 (1.1%)	920 (0.8%)	978 (0.3%)	502 (0.5%)
Unknown	44,381 (8.5%)	6,079 (5.5%)	19,884 (6.5%)	4,905 (4.7%)
Charlson Comorbidity Index				
0	194,689 (37.3%)	46,596 (41.8%)	126,679 (41.4%)	44,822 (42.9%)
1-2	188,703 (36.2%)	42,488 (38.1%)	119,336 (39.0%)	40,479 (38.7%)
3-4	54,101 (10.4%)	11,228 (10.1%)	29,086 (9.5%)	9,914 (9.5%)
5-10	32,788 (6.3%)	5,513 (4.9%)	15,113 (4.9%)	4,648 (4.4%)
11+	3,644 (0.7%)	523 (0.5%)	638 (0.2%)	296 (0.3%)
Missing	47,454 (9.1%)	5,095 (4.6%)	15,280 (5.0%)	4,351 (4.2%)
Community Wellbeing Index^3^				
0-45	2,646 (0.5%)	275 (0.2%)	327 (0.1%)	157 (0.2%)
46-55	152,670 (29.3%)	26,978 (24.2%)	91,926 (30.0%)	25,470 (24.4%)
56-65	193,631 (37.1%)	46,639 (41.9%)	106,456 (34.8%)	43,891 (42.0%)
66-100	26,644 (5.1%)	9,961 (8.9%)	13,750 (4.5%)	8,879 (8.5%)
Missing	145,788 (28.0%)	27,590 (24.8%)	93,673 (30.6%)	26,113 (25.0%)
Month of COVID-19 diagnosis				
December 2021	39,670 (7.6%)	77 (0.1%)	-	-
January 2022	166,565 (31.9%)	1,544 (1.4%)	902 (0.3%)	64 (0.1%)
February 2022	37,600 (7.2%)	931 (0.8%)	69,739 (22.8%)	1,495 (1.4%)
March 2022	11,775 (2.3%)	845 (0.8%)	16,468 (5.4%)	865 (0.8%)
April 2022	16,902 (3.2%)	3,805 (3.4%)	4,226 (1.4%)	725 (0.7%)
May 2022	38,567 (7.4%)	12,984 (11.7%)	10,782 (3.5%)	3,420 (3.3%)
June 2022	39,959 (7.7%)	14,950 (13.4%)	31,292 (10.2%)	12,227 (11.7%)
July 2022	45,957 (8.8%)	19,455 (17.5%)	32,867 (10.7%)	14,135 (13.5%)
August 2022	39,646 (7.6%)	16,128 (14.5%)	39,158 (12.8%)	18,580 (17.8%)
September 2022	26,454 (5.1%)	11,101 (10.0%)	32,965 (10.8%)	15,303 (14.6%)
October 2022	20,185 (3.9%)	8,386 (7.5%)	20,819 (6.8%)	10,338 (9.9%)
November 2022	18,149 (3.5%)	9,131 (8.2%)	15,889 (5.2%)	7,766 (7.4%)
December 2022	19,950 (3.8%)	12,106 (10.9%)	14,412 (4.7%)	8,275 (7.9%)

1 Any hospital admission in the 28-days following a positive SARS-CoV-2 test result

2 *abbrev*. NH = Non-Hispanic.

3 Community Wellbeing Index (CWBI) is a measure of five inter-related community-level domains, with higher CWBI values corresponding to a higher level of protective community-level social determinants of health. ^32^

**Table 3:** Odds of hospitalization in Paxlovid-treated vs. Non-Paxlovid-treated patients

	*Dependent variable:* Hospitalization		
	Unadjusted (1)	Adjusted (2)	Vaccine-adjusted (3)
	OR (95% CI)	OR (95% CI)	OR (95% CI)
Paxlovid Treatment			
No (control)	*ref*	*ref*	*ref*
Yes (treatment)	0.33 (0.24-0.45)***	0.35 (0.29-0.42)***	0.32 (0.24-0.42)***
Vaccination Status^1^			
Unvaccinated	-	-	*ref*
Vaccinated	-	-	0.49 (0.41-0.58)***
Vaccination*Paxlovid^2^	-	-	1.08 (0.71-1.64)

*Observations*	410,642	410,642	136,815
*Log Likelihood*	−31590.1	−25466.1	−6551.1
*Akaike Inf. Crit*.	63184.0	51000.2	13172.3

1 Unvaccinated refers to patients who received 0 doses at index, Vaccinated refers to patients who recieved atleast 2 doses at least 14 days prior to index.

2 Interaction term

Note: Model (2) adjusts for sex, age, race and ethnicity, Charlson Comorbidity Index, Community-well being index, data partners, and month of COVID-19 Index date. Model (3) additionally adjusts for the main and interaction of Vaccination.

* *p* < .05

** *p* < .01,

*** *p* < .001

## Data Availability

All data is available in the N3C Data Enclave to those with an approved protocol and data use request from an institutional review board. Data access is governed under the authority of the National Institutes of Health; more information on accessing the data can be found at https://covid.cd2h.org/for-researchers. See Haendel et. al. for additional detail on how data is ingested, managed, and protected within the N3C Data Enclave.^34^
