# Supplementary material for: Effect of Nirmatrelvir/Ritonavir (Paxlovid) on Hospitalization among Adults with COVID-19: an EHR-based Target Trial Emulation from N3C
Source: medRxiv. 2023 May 4:2023.05.03.23289084. Preprint. [Version 1] doi: 10.1101/2023.05.03.23289084 (PMC10187454; doi:10.1101/2023.05.03.23289084)
Supplement: 1 [file NIHPP2023.05.03.23289084v1-supplement-1.pdf]

medRxiv preprint doi: https://doi.org/10.1101/2023.05.03.23289084; this version posted May 4, 2023. The copyright holder for this preprint (which was not certified by peer review) is the author/funder, who has granted medRxiv a license to display the preprint in perpetuity.
It is made available under a CC-BY 4.0 International license .

Supplementary Tables and Figures

Fig. 1 Proportion of individuals in base population stratified by Race/Ethnicity, and ZIP Code-level Community Wellbeing Index (CWBI)

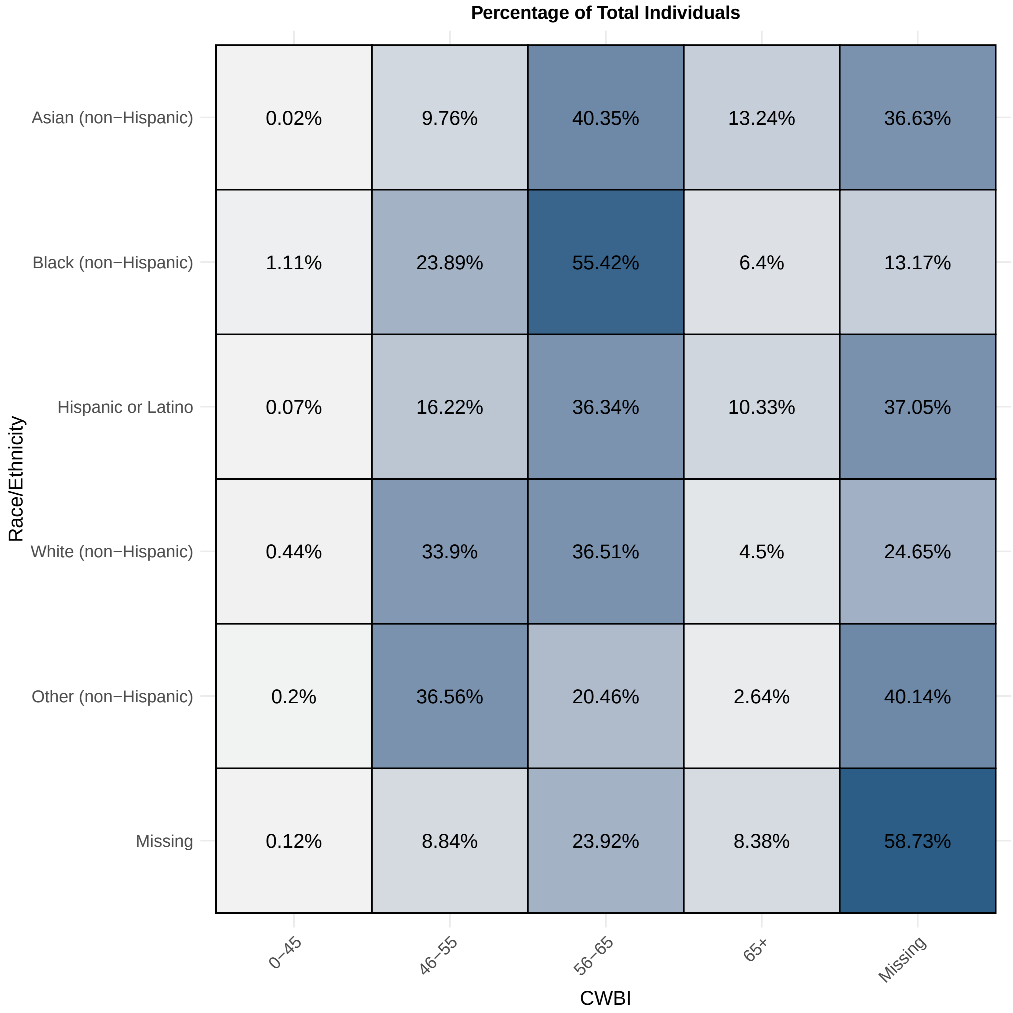

medRxiv preprint doi: https://doi.org/10.1101/2023.05.03.23289084; this version posted May 4, 2023. The copyright holder for this preprint (which was not certified by peer review) is the author/funder, who has granted medRxiv a license to display the preprint in perpetuity.
It is made available under a CC-BY 4.0 International license .

Table 1 Sensitivity analysis examining the effects of immortal time bias, and missingness in the ZIP Code-level Community Wellbeing Index (CWBI)

	Dependent variable: Hospitalization		
	Not accounting for Immortal Time Bias	Dropping CWBI covariate	Dropping sites with missing CWBI
	OR (95% CI)	OR (95% CI)	OR (95% CI)
Paxlovid Treatment			
No (control)	*ref*	*ref*	*ref*
Yes (treatment)	0.38 (0.31-0.46)***	0.37 (0.31-0.45)***	0.37 (0.29-0.47)***
Observations	410,716	455,504	272,133
Log Likelihood	-29151.5	-29618.4	-20744.2
Akaike Inf. Crit.	58376.9	59296.7	41562.4

Note: $p < .05$*, $p < .01$**, $p < .001$***
